# Identification and characterization of long non-coding RNAs in porcine granulosa cells exposed to 2,3,7,8-tetrachlorodibenzo-*p*-dioxin

**DOI:** 10.1186/s40104-018-0288-3

**Published:** 2018-10-11

**Authors:** Monika Ruszkowska, Anna Nynca, Lukasz Paukszto, Agnieszka Sadowska, Sylwia Swigonska, Karina Orlowska, Tomasz Molcan, Jan P. Jastrzebski, Renata E. Ciereszko

**Affiliations:** 1 Department of Animal Anatomy and Physiology, Faculty of Biology and Biotechnology, University of Warmia and Mazury in Olsztyn, Oczapowskiego 1A, 10-719 Olsztyn, Poland; 2 Laboratory of Molecular Diagnostics, Faculty of Biology and Biotechnology, University of Warmia and Mazury in Olsztyn, Prawochenskiego 5, 10-720 Olsztyn, Poland; 3 Department of Plant Physiology, Genetics and Biotechnology, Faculty of Biology and Biotechnology, University of Warmia and Mazury in Olsztyn, Oczapowskiego 1A, 10-719 Olsztyn, Poland

**Keywords:** AVG-16 cell line, Granulosa cells, lncRNAs, Pig, RNA-Seq, TCDD

## Abstract

**Background:**

Long non-coding RNAs (lncRNAs) may regulate gene expression in numerous biological processes including cellular response to xenobiotics. The exposure of living organisms to 2,3,7,8-tetrachlorodibenzo-*p*-dioxin (TCDD), a persistent environmental contaminant, results in reproductive defects in many species including pigs. The aims of the study were to identify and characterize lncRNAs in porcine granulosa cells as well as to examine the effects of TCDD on the lncRNA expression profile in the cells.

**Results:**

One thousand six hundred sixty-six lncRNAs were identified and characterized in porcine granulosa cells. The identified lncRNAs were found to be shorter than mRNAs. In addition, the number of exons was lower in lncRNAs than in mRNAs and their exons were longer. TCDD affected the expression of 22 lncRNAs (differentially expressed lncRNAs [DELs]; log_2_ fold change  ≥ 1, *P-adjusted* < 0.05) in the examined cells. Potential functions of DELs were indirectly predicted via searching their target *cis*- and *trans*-regulated protein-coding genes. The co-expression analysis revealed that DELs may influence the expression of numerous genes, including those involved in cellular response to xenobiotics, dioxin metabolism, endoplasmic reticulum stress and cell proliferation. Aryl hydrocarbon receptor (*AhR)* and cytochrome P450 1A1 (*CYP1A1)* were found among the *trans*-regulated genes.

**Conclusions:**

These findings indicate that the identified lncRNAs may constitute a part of the regulatory mechanism of TCDD action in granulosa cells. To our knowledge, this is the first study describing lncRNAs in porcine granulosa cells as well as TCDD effects on the lncRNA expression profile. These results may trigger new research directions leading to better understanding of molecular processes induced by xenobiotics in the ovary.

**Electronic supplementary material:**

The online version of this article (10.1186/s40104-018-0288-3) contains supplementary material, which is available to authorized users.

## Background

Recent advances in RNA sequencing (RNA-Seq) technologies led to the discovery of tens of thousands non-coding RNAs (ncRNAs) across the entire transcriptomes of many species. Previously, ncRNAs were considered as evolutionary junk or transcriptional noise, but recently they were reported to play a crucial role in the regulation of gene expression [1, 2]. The class of ncRNAs includes short ncRNAs (e.g., microRNA, piwiRNA), housekeeping ncRNAs (e.g., ribosomal RNA, transfer RNA) as well as long non-coding RNAs (lncRNAs), which constitute the largest group of ncRNAs [3, 4]. In broad terms, lncRNAs are defined as non-coding transcripts longer than 200 nucleotides (nt). Similar to mRNAs, most known lncRNAs: 1) are transcribed by RNA polymerase II, 2) contain an intron-exon structure and 3) undergo post-transcriptional modifications, e.g., 5′ capping, 3′ polyadenylation or alternative splicing [5]. lncRNAs originate from intergenic regions of the genome (lincRNAs), introns of protein-coding genes (linRNAs) or the opposite strand of DNA (antisense lncRNAs) [5, 6]. It was demonstrated that lncRNA sequences are not highly conserved and their expression profiles differ among species, developmental stages and cell types. Due to their variable nature, lncRNAs are still poorly characterized [7, 8].

It was found that lncRNAs regulate gene expression via numerous mechanisms. lncRNAs may be implicated in e.g., chromatin organization, epigenetic modification, transcriptional regulation, RNA processing and modification, regulation of miRNA activity as well as protein stability or cellular protein localization [3, 8, 9]. lncRNAs seem to be involved in many fundamental biological processes including maintenance and induction of stem cell pluripotency, cell differentiation, cell proliferation and apoptosis [6, 8]. The mutations of lncRNAs and deregulation of lncRNA expression have been associated with a number of human diseases, such as different types of cancer as well as neurobiological, cardiovascular and autoimmune disorders [6, 8]. The results of recent studies also indicate that lncRNAs take an active part in cellular response to various xenobiotics [1].

2,3,7,8-tetrachlorodibenzo-*p*-dioxin (TCDD), a chlorinated xenobiotic and a member of the polycyclic aromatic hydrocarbon (PAH) family, is a by-product of human activity including herbicide production, waste incineration and fossil fuel burning [10]. Because of TCDD lipid solubility and high resistance to degradation, its half-life is long and ranges from 7 to 10 years in human bodies and from 25 to 100 years in the environment. As a result, TCDD persists in soil and water sediments as well as in plant and animal organisms [11, 12]. The main intracellular mechanism of TCDD action involves the activation of the aryl hydrocarbon receptor (AhR) pathway. AhR is a ligand-activated transcription factor and a member of the basic helix-loop-helix/PER-ARNT-SIM (bHLH-PAS) family. Upon activation, the ligand-AhR complex translocates to the nucleus, dimerizes with AhR nuclear translocator (ARNT) and induces the expression of different genes including xenobiotic-metabolizing enzymes [13]. Exposure to TCDD may result in a variety of harmful short- and long-term effects, such as wasting syndrome, cancer and neurological dysfunctions. TCDD has also been demonstrated to cause endocrine disruption and reproductive defects in many species including pigs [14–19].

Granulosa cells play a critical role in maintaining ovarian function and, in consequence, female fertility. These cells protect and nurture oocytes as well as produce steroid hormones (estradiol [E_2_], progesterone [P_4_]) responsible for creating an optimal environment for follicular development, fertilization, implantation and embryo growth [20]. Due to the AhR presence in porcine granulosa cells [21, 22] and the fact that TCDD affects granulosal production of E_2_ and P_4_ in pigs [14, 15, 19], it is necessary to indicate molecular targets of TCDD in these cells. The effects of TCDD on gene expression in porcine granulosa cells were described in our recent paper [23], but information concerning lncRNAs in the porcine ovary is scarce [24]. We assumed that the transcriptome of porcine granulosa cells contains lncRNAs and that some lncRNAs are involved in TCDD action in the cells. Therefore, the aims of the current study were to: 1) identify and characterize porcine granulosa lncRNAs, 2) examine the effects of TCDD on the lncRNA expression profile (i.e., to identify differentially expressed lncRNAs [DELs]) and 3) predict target genes for DELs in order to tentatively evaluate their regulatory role in granulosal response to TCDD.

## Methods

### Culture of AVG-16 cells

AVG-16 cell line originating from granulosa cells of medium porcine follicles was used in the current study (06062701; The European Collection of Authenticated Cell Cultures; UK; [25]). Previously, we have showed that AVG-16 cells are morphologically and physiologically similar to primary porcine granulosa cells and are a good model for studying xenoestrogen effects on ovarian functions [22]. Before the experiment, the cells were thawed and cultured in six-well plates with seeding density of 1 × 10^6^ cells/3 mL culture medium (Dulbecco’s modified Eagle’s medium [DMEM] with 2 mmol/L *L*-glutamine, 10% fetal bovine serum [FBS], 0.1 mmol/L non-essential amino acid [NEAA], 2.5 ng/mL fibroblast growth factor-basic human [bFGF] and antibiotic mixture [100 U penicillin, 100 μg streptomycin and 0.25 μg amphotericin B/mL]; Sigma Aldrich, St. Louis, MO, USA) [22, 23]. After reaching 60–65% confluency, the cells were treated with TCDD (100 nmol/L; Sigma Aldrich) for 3, 12 or 24 h (*n* = 2 biological replicates per one time point). To bring out the potential of TCDD to transduce intracellular signaling in the examined cells, the selected dose of TCDD moderately exceeded its environmentally relevant concentration. Moreover, 100 nmol/L of TCDD was found to be effective in porcine granulosa cells (steroidogenesis: [26–28]; gene expression: [23, 29]). At the end of the culture, medium was removed, cells were washed twice with a phosphate-buffered saline (PBS; Sigma Aldrich) and designated for total RNA isolation.

### Total RNA isolation, evaluation of RNA integrity, cDNA library construction and sequencing

Total RNA was isolated from cells using peqGold TriFast (Peqlab Biotechnologie GmbH, Erlangen, Germany) according to manufacturer’s instructions. RNA concentration and quality were determined spectrophotometrically (NanoVue Plus, GE Healthcare, Little Chalfont, UK). The integrity of total RNA was assessed by Agilent 2100 Bioanalyzer using RNA 6000 Nano LabChip Kit (Agilent Technologies, Santa Clara, CA, USA). Only samples with RNA integrity number (RIN) values higher than 8.0 were used for RNA-Seq.

Depleted RNA obtained from 400 ng of total RNA was used to construct cDNA libraries (TruSeq RNA Sample Preparation Kit; Illumina, San Diego, CA, USA). Following RNA purification and fragmentation, first and second cDNA strands were synthesized. Next steps included 3’ end adenylation, adapter ligation and library amplification (PCR). Quantification of the cDNA library templates was performed using KAPA Library Quantification Kit (Kapa Biosystem, Wilmington, MA, USA). Library profiles were estimated with the use of DNA High Sensitivity LabChip Kit (Agilent Technologies) and 2100 Bioanalyzer. The cDNA library templates were sequenced using HiSeq 2500 high throughput sequencing instrument (Illumina) with 100 paired-end sequencing. The RNA-Seq analyses of protein-coding genes were described in a separate manuscript [23]. This article is focused on the identification and characterization of long non-coding RNAs in porcine granulosa cells exposed to TCDD.

### Sequencing data analysis and transcriptome assembly

The sequencing data from this study have been submitted to the NCBI BioProject database (http://www.ncbi.nlm.nih.gov/bioproject) under accession number: PRJNA429720. The quality of cDNA fragments obtained after sequencing (raw reads) was first evaluated using FastQC program (http://www.bioinformatics.babraham.ac.uk/projects/fastqc/). Next, the raw reads were trimmed (Trimmomatic tool version 0.32) by removing the adapter sequences and reads shorter than 90 nt [30]. After trimming to the same length (90 nt), the fragments were mapped to the porcine reference genome (Sus_scrofa.Sscrofa10.2; Ensembl database) using STAR version 2.4 [31]. The mapped reads were assembled into contigs with Cufflinks version 2.2.1 [32] and StringTie version 1.0.4 [33, 34].

### Identification of lncRNAs

**Fig. 1:**
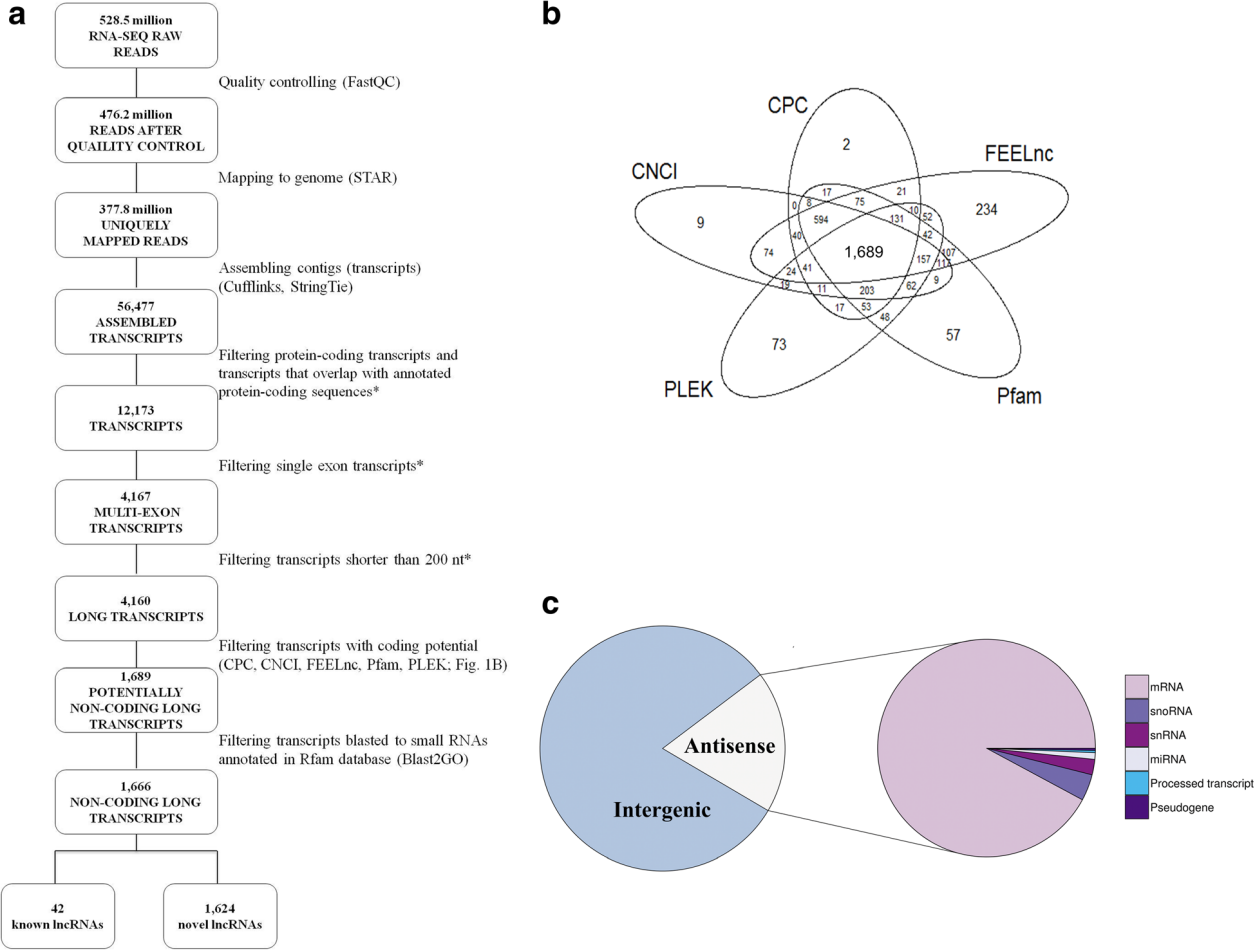
Screening of candidate long non-coding RNAs (lncRNAs) in the porcine granulosa cell transcriptome and classification of novel lncRNAs. **a** Schematic diagram of the pipeline used for the identification of lncRNAs in porcine granulosa cells. **b** Venn diagram presenting the results of the coding potential analysis of the obtained 4,160 long transcripts. Please note that five different tools (CPC, CNCI, PLEK, Pfam, FEELnc) were employed to analyze the coding potential. In consequence, 1,689 potentially non-coding long transcripts were identified and designated as candidate lncRNAs. **c** Classification of the obtained novel lncRNAs according to their genomic positions. *Customized algorithms of the authors were applied for the lncRNA identification in this step

A customized multi-step pipeline (Fig. 1a) was employed to identify putative novel lncRNAs in porcine granulosa cells. The detailed identification steps included removal of: 1) protein-coding transcripts and transcripts that overlap, in sense orientation, with at least one base of all porcine protein-coding sequences annotated in the Ensembl database; 2) transcripts with a single exon [35, 36]; 3) transcripts shorter than 200 nt; 4) transcripts which were predicted by Coding Potential Calculator (CPC) (score < 0) [37], Coding-Non-Coding Index (CNCI) (score < 0) [38], FlExible Extraction of LncRNAs (FEELnc) (coding potential > 0.558) [39], Pfam Scan (v1.3) (*E*-value < 0.001) [40] and PLEK (score < 0) [41] to encode proteins and 5) transcripts blasted (Blast2GO) to small RNAs (rRNA, tRNA, snRNA, snoRNA, miRNA, etc.) annotated in Rfam database. The remaining transcripts were considered as lncRNAs and were divided into known lncRNAs (based on the pig Ensembl database lncRNAs annotation) and novel lncRNAs. In addition, the obtained lncRNAs were classified depending on their genomic locations. The protein-coding sequences removed in step 1 of the identification pipeline were characterized and their genomic features were compared with those of lncRNAs (Welch’s *t*-test in R package, *P* < 0.05).

### Differential expression analysis of lncRNAs

The combined Cufflinks and StringTie tools allowed us to analyze the expression profile of both known and unannotated lncRNAs. The identified lncRNA sequences were normalized to FPKM (fragments per kilobase of exon per million fragments mapped) values using the Cuffnorm (version 2.2.1) in the Cufflinks package [32]. To identify differentially expressed lncRNAs (DELs), the expression levels of lncRNAs in TCDD-treated cells were compared to their respective expression levels in control cells. The corresponding *P*-values were determined for each incubation time by means of Cuffdiff [32]. *P-adjusted* < 0.05 and log_2_ fold change (log_2_FC) ≥ 1.0 were set as a threshold for the significantly different expression.

### *Cis*- and *trans*-regulated target gene prediction and Gene Ontology (GO) enrichment analysis

*Cis*-acting lncRNAs regulate the expression of genes that are positioned in the vicinity of their transcription sites, whereas *trans*-acting lncRNAs modulate the expression of genes being at independent loci [42]. Protein-coding genes located within 10 kb from DELs were screened with the use of R package [43] and selected as DEL potential *cis*-regulated targets [44, 45]. To explore the *trans*-type interaction, the Pearson’s correlations coefficients (*r* > 0.7 or *r* < − 0.7) between DELs and protein-coding genes were analyzed. To analyze functions of the potential DEL target genes and their involvement in biological processes, the GO database was used (the established criteria: *P-adjusted* < 0.05). GO enrichment analysis was performed using g:Profiler software [46].

### Alternative splicing events analysis

To recognize the differential expression of exons and the occurrence of splice junctions (i.e., differential usage of exons and junctions) in the identified lncRNAs, QoRTs [47] and JunctionSeq [48] tools were applied. First, QoRTs was used to generate the raw exon- and splice junction-counts. Then, JunctionSeq was employed to visualize the identified sites of differential usage of exons as well as splice junction in lncRNA loci of TCDD-treated cells in comparison to control cells (*P-adjusted* < 0.05).

### Real-time PCR

To validate RNA-Seq results, three lncRNAs, expression of which was affected by TCDD in two (TCONS_00016901; TCONS_00031035) or three (TCONS_00034713) incubation times, were selected for real-time PCR (qRT-PCR). Complementary DNA was synthesized using RNA isolated from granulosa cells (control and TCDD-treated), 0.5 μmol/L oligo(dT)_15_ primer (Roche, Basel, Switzerland), 1 μmol/L hexanucleotide primers, 10 U RNase Out (Sigma Aldrich) and Omniscript RT Kit (Qiagen, Hilden, Germany). Reverse transcription reaction was performed at 37 °C for 1 h (Veriti Thermal Cycler, Thermofisher Scientific, Waltham, MA, USA). Specific primers for particular lncRNA were synthesized by Thermofisher Scientific Company (TCONS_00031035 – Assay ID: AIPAFN7; TCONS_00016901 – Assay ID: AIN1HH2; TCONS_00034713 – Assay ID: AIFAT9T). Glyceraldehyde 3-phosphate dehydrogenase (*GAPDH*; Assay ID: Ss03373286_u1) and β-actin (Assay ID: Ss03376563_uH) were used as reference genes. Real-time PCR was performed using TaqMan® Universal PCR Master Mix and TaqMan Gene Expression Assay (Thermofisher Scientific) in Applied Biosystems 7500 Fast Real-Time PCR System (Thermofisher Scientific). Thermal cycling conditions consisted of an initial denaturation step at 95 °C for 10 min and then 40 cycles of denaturation at 95 °C for 15 s followed by primer annealing at 60 °C for 1 min. The qRT-PCR for each sample was carried out in duplicate and non-template control was included in each run. To present the data as arbitrary units of the relative expression, lncRNA expression levels were normalized to the expression of *GAPDH* and *β-actin*. This was done by using comparative cycle threshold (Ct) method and the quantity based active schematic estimating (Q-BASE) model [49]. Data were expressed as mean ± SEM. The difference in lncRNA expression level between control and TCDD-treated cells was evaluated using Student’s *t*-test (Statistica Software Inc., Tulsa, OH, USA). Differences with a probability of *P* < 0.05 were considered significant.

## Results

The sequencing of the porcine granulosa cell transcriptome provided 528.5 million reads (50–100 nt). After discarding adaptor sequences and low-quality sequences (Phred score Q < 20), the remaining 476.2 million reads were mapped to the annotated whole porcine genome (Sus_scrofa.Sscrofa10.2). The percentage of unique mapped reads ranged from 76.8% to 80.1%. As a result, 56,477 transcripts were collected (see Additional file 1).

### Identification of lncRNAs in the porcine granulosa cell transcriptome

To identify lncRNAs from 56,477 mapped transcripts, the customized multi-step identification pipeline was applied (Fig. 1a). Twelve thousand one hundred seventy-three transcripts were obtained after filtering the protein-coding transcripts and transcripts that overlap with annotated protein-coding sequences. The removal of single exon transcripts and transcripts shorter than 200 nt yielded 4,160 transcripts and the evaluation of the protein-coding potential (Fig. 1b) produced 1,689 potentially non-coding long transcripts. The stringency of the selection process was additionally increased by discarding transcripts which blasted to small RNAs annotated in the Rfam database. Eventually, 1,666 RNA sequences were identified as lncRNAs, and 42 of them have already been annotated in databases. According to genomic localization of lncRNAs, the identified porcine novel lncRNAs were classified as intergenic lncRNAs (1,319 transcripts) and antisense lncRNAs (305 transcripts). The group of antisense lncRNAs (Fig. 1c) were located at the opposite strand of annotated protein-coding genes (281 lncRNAs), snoRNAs (12 lncRNAs), snRNAs (7 lncRNAs), miRNAs (3 lncRNAs), processed transcript (1 lncRNA) and pseudogene (1 lncRNA).

### The comparison of lncRNAs and mRNAs of the granulosa cell transcriptome

**Fig. 2:**
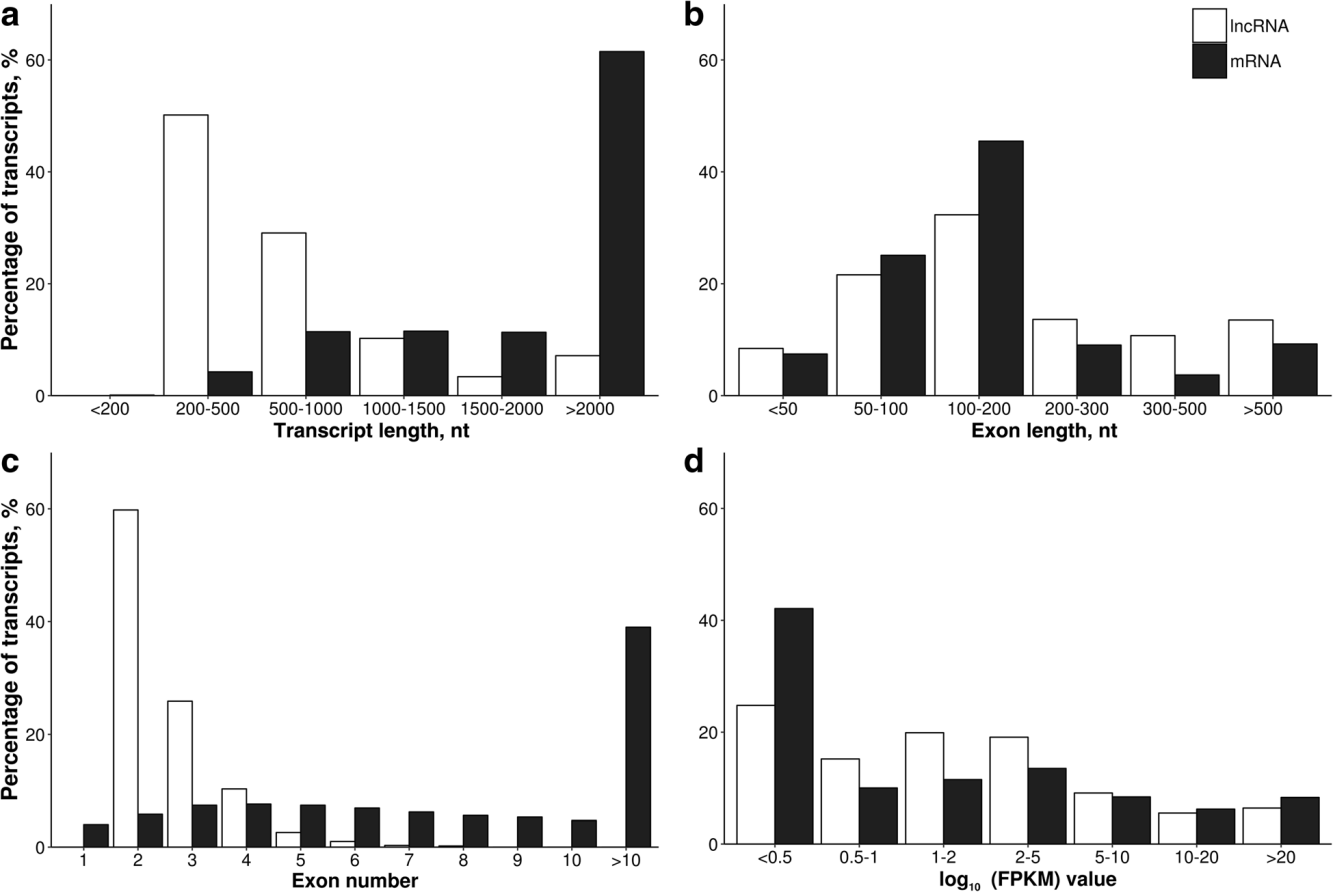
The percentage distribution of the identified lncRNAs and mRNAs according to their (**a**) transcript length, (**b**) exon length, (**c**) exon number and (**d**) expression level (the latter expressed as fragments per kilobase of exon per million fragments mapped [FPKM] values). The assumed nucleotide (nt) ranges as well as ranges of FPKM values are depicted in the *X* axis

The transcript length, exon length, exon number and expression level were compared between the identified lncRNAs (1,666 transcripts) and mRNAs (42,913 transcripts). The length of most of the lncRNAs ranged from 200 to 1,000 nt (Fig. 2a), while the length of most lncRNA exons ranged from 50 to 200 nt (Fig. 2b). Moreover, a majority of lncRNAs consisted of two exons (Fig. 2c) and the expression level of more than 50% of lncRNAs was lower than FPKM value of 2 (Fig. 2d).

**Fig. 3:**
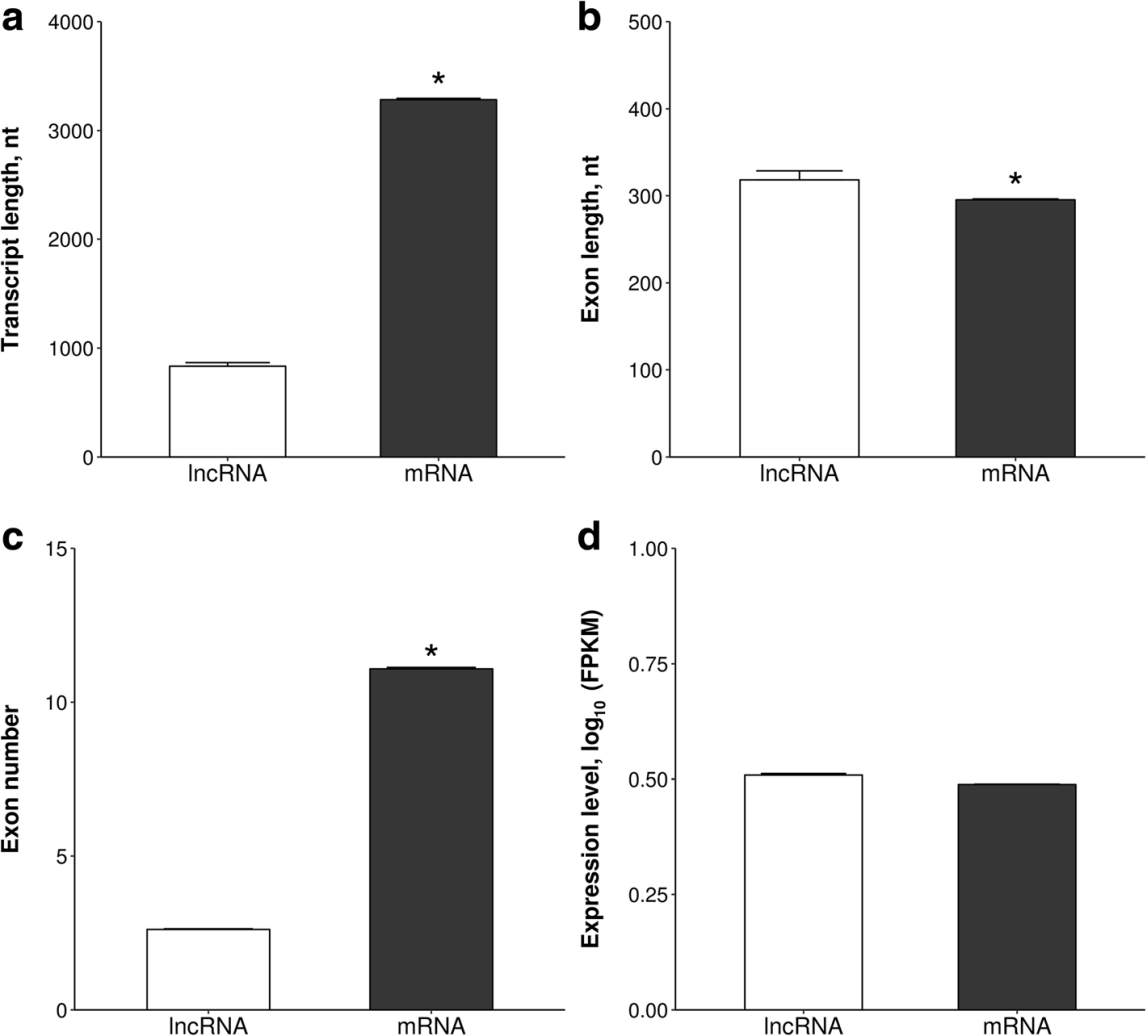
The comparison of genomic features of the identified lncRNAs and mRNAs. The lncRNAs and mRNAs were compared in respect to average (**a**) transcript length, (**b**) exon length, (**c**) exon number and (**d**) expression level. Data are expressed as mean ± SE. Statistical analysis was performed using Welch’s *t*-test in R package. Asterisks designate statistical differences (*P* < 0.05)

The average length of lncRNAs (832.6 ± 33.06 nt) was shorter (*P* < 0.05) than that of mRNA (3284.4 ± 12.49 nt) (Fig. 3a) and the average exon length of lncRNA (318.2 ± 10.39 nt) was longer (*P* < 0.05) than that of mRNA (295.3 ± 1.03 nt) (Fig. 3b). In addition, the mean exon number of lncRNAs (2.6 ± 0.02) was lower (*P* < 0.05) than the mean exon number of mRNAs (11.09 ± 0.05) (Fig. 3c). The mean expression level (0.51 ± 0.003 vs. 0.49 ± 0.001 FPKM) did not differ (*P* > 0.05) between lncRNA and mRNA (Fig. 3d).

### Differentially expressed lncRNAs in TCDD-treated porcine granulosa cells

**Fig. 4:**
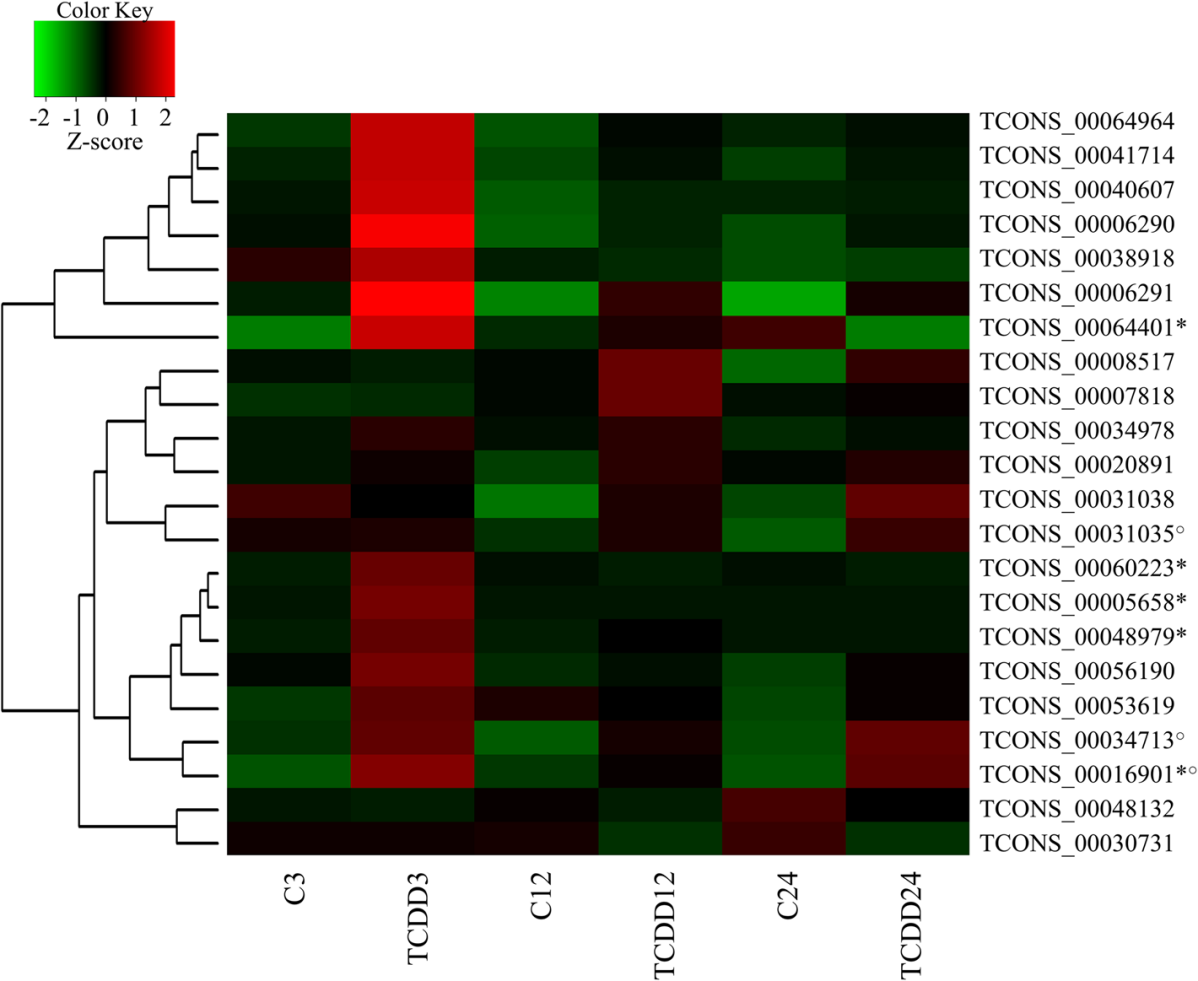
Heatmap illustrating the expression profile of differentially expressed lncRNAs (presented as Z-score values) in porcine granulosa cells treated with TCDD for 3, 12 or 24 h. Red blocks represent up- and green blocks represent down-regulated lncRNAs. The color scale of the heatmap shows the expression level where the brightest green stands for − 2.0 Z-score and the brightest red stands for + 2.0 Z-score. Z-score was calculated using logFPKM and the following equation: [*x* - mean(*x*)] / sd(*x*) where ‘sd’ means standard deviation. C 3–24: untreated porcine granulosa cells (control) cultured for 3, 12 or 24 h. TCDD 3–24: porcine granulosa cells treated with TCDD (100 nmol/L) for 3, 12 or 24 h. *transcript expression was not detected in control (untreated) cells; ○ transcripts with expression level altered in two or three examined incubation times

Twenty two differentially expressed lncRNAs (*P-adjusted* < 0.05 and log_2_FC ≥ 1.0) were identified. The expression of 15, 4 and 7 lncRNAs were significantly altered after 3, 12 and 24 h of TCDD treatment, respectively. The log_2_FC value for DELs ranged from 3.77 (TCONS_00041714) to − 1.01 (TCONS_00048132) (Table 1). Among all DELs: 1) one lncRNA (TCONS_00034713) was up-regulated by TCDD in all incubation times, 2) two lncRNAs (TCONS_00016901 and TCONS_00031035) were up-regulated by the dioxin after two incubation times (3, 24 and 12, 24 h of TCDD treatment, respectively), 3) two lncRNAs (TCONS_00048132; TCONS_00030731) were down-regulated, and the downregulation was found after 24 h of TCDD treatment, 4) five lncRNAs (TCONS_00005658; TCONS_00016901; TCONS_00048979; TCONS_00060223; TCONS_00064401) were expressed only in TCDD-treated cells and 5) three lncRNAs (TCONS_00038918, TCONS_00030731, TCONS_00064964) up-regulated by TCDD were located in the antisense strand of protein-coding transcripts (MAF bZIP transcription factor F [*MafF*], transforming growth factor beta-induced [*TGFβI*], NHS actin remodeling regulator [*NHS*]). The expression profile of up- and down-regulated lncRNAs identified in granulosa cells after 3, 12 or 24 h of TCDD treatment is presented in Fig. 4.

**Table 1:** Differentially expressed lncRNAs (log_2_FC ≥ 1)^a^ identified in porcine granulosa cells after 3, 12 or 24 h of TCDD treatment

No.	Identified lncRNA	lncRNA XLOC	lncRNA locus	log_2_FC		
				3 h	12 h	24 h
1	TCONS_00005658	XLOC_002986	10:777270–897286	Inf^b^	–	–
2	TCONS_00006290	XLOC_003288	7:63840034–63853541	3.34	–	–
3	TCONS_00006291	XLOC_003288	10:78713507–78719836	3.32	–	–
4	TCONS_00016901	XLOC_008756	14:120019330–120022623	Inf	–	Inf
5	TCONS_00034713	XLOC_017807	3:108221745–108230820	1.76	1.64	2.32
6	TCONS_00034978	XLOC_017941	3:138550166–138568232	1.16	–	–
7	TCONS_00038918	XLOC_019982	5:6915996–7133894	1.34	–	–
8	TCONS_00040607	XLOC_020835	5:23100502–23101339	2.07	–	–
9	TCONS_00041714	XLOC_021448	6:3237107–3238658	3.77	–	–
10	TCONS_00048979	XLOC_024965	7:3272029–3276056	Inf	–	–
11	TCONS_00053619	XLOC_027505	9:32511923–32566577	1.51	–	–
12	TCONS_00056190	XLOC_028876	9:146755972–146762016	2.08	–	–
13	TCONS_00060223	XLOC_031885	GL895718.2:3829–4611	Inf	–	–
14	TCONS_00064401	XLOC_034330	X:123166327–123167560	Inf	–	–
15	TCONS_00064964	XLOC_034502	X:14951320–15305754	3.40	–	–
16	TCONS_00007818	XLOC_004159	11:18740477–18860373	–	1.09	–
17	TCONS_00020891	XLOC_010641	15:71337144–71348412	–	0.99	–
18	TCONS_00031035	XLOC_015924	2:160084074–160102520	–	1.34	2.68
19	TCONS_00008517	XLOC_004581	12:6857198–6863409	–	–	2.06
20	TCONS_00030731	XLOC_015756	2:143856229–143907415	–	–	−0.99
21	TCONS_00031038	XLOC_015924	2:160084074–160102520	–	–	2.31
22	TCONS_00048132	XLOC_024540	7:63840034–63853541	–	–	−1.01

^a^log_2_FC – log_2_ fold change

^b^Inf – transcript expression was not detected in control (untreated) cells

### The *cis*- and *trans*-target genes for DELs

The potential target genes for DELs acting in a *cis*- or *trans*-regulatory manner were predicted to investigate the possible significance of these lncRNAs in the porcine granulosal response to TCDD. In silico analysis produced 10 *cis*-target genes for DELs (see Additional file 2) and none of them were enriched (*P* > 0.05) in the GO classification.

To analyze the *trans-*type interaction between DELs (Table 1) and their target genes, the co-expression analysis was performed. As a result, a total of 916 negative (see Additional file 3) and 1,143 positive (see Additional file 4) correlations were detected. The negatively co-expressed genes were enriched in 28 GO terms (26 under “biological process” and two under “molecular function”) including “intracellular signal transduction” (GO:0035556), “response to stimulus” (GO:0050896) and “negative regulation of biological process” (GO:0048519). The negatively co-expressed *trans*-target genes involved, among others, *AhR*, *TGFβI*, endoplasmic reticulum to nucleus signaling 1 (*ERN1*), LIM domain kinase 2 (*LIMK 2*) and ephrin type-B receptor 4 (*EPHB4*) (see Additional file 5). The positively co-expressed genes were enriched in five GO terms (four in “biological process” and one in “molecular function”). Some of the genes were related to cellular response to xenobiotics, including “cellular response to chemical stimulus” (GO:0070887) and “regulation of signal transduction” (GO:0009966). This group of *trans*-target genes involved cytochrome P4501A1 (*CYP1A1*), *MafF* and *NHS* (see Additional file 6).

### Alternative splicing events of lncRNAs in TCDD-treated porcine granulosa cells

**Fig. 5:**
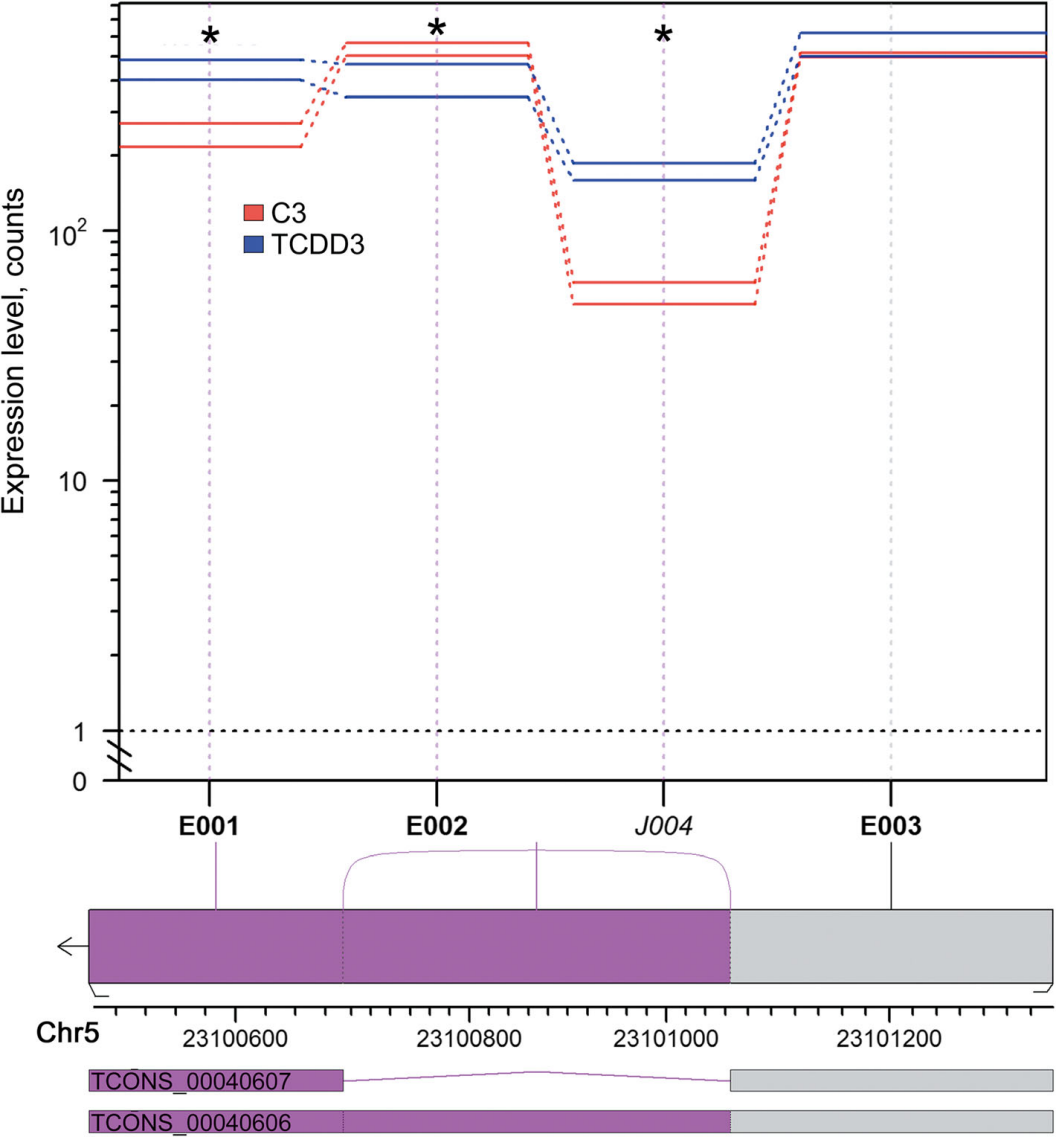
Presentation of differential expression of exons (E) and the occurrence of splice junction (J) in the exemplary selected lncRNA XLOC (XLOC_020835) identified in porcine granulosa cells treated with TCDD. The upper panel shows the expression level estimates for the mean normalized read counts for each exon or splice junction of XLOC_020835 identified in TCDD-treated (blue) and control (red) cells. The lower panel shows the exonic regions (boxes, labelled E001-E003) and known splice junctions (solid line, labelled J004). Statistically significant differences (*P-adjusted* < 0.05) of usage exons and junctions are drawn in violet. C3: untreated porcine granulosa cells (control) cultured for 3 h. TCDD3: porcine granulosa cells treated with TCDD (100 nmol/L) for 3 h

We identified 33 events (sites) of differential usage of exons and splice junctions in lncRNAs loci of porcine granulosa cells after 3 h of TCDD treatment and only four events after 12 h of the treatment. No differential usage of exons and junctions was found after 24 h of TCDD treatment. Among the identified events, 28 and 2 were defined as differentially expressed exons, whereas 5 and 2 were described as splice junctions after 3 h and 12 h of TCDD treatment, respectively (see Additional file 7). The JunctionSet profile plot for the exemplary lncRNA XLOC (XLOC_020835) is presented in Fig. 5. Two lncRNAs - TCONS_00040606 and TCONS_00040607 – may be formed on the basis of this selected XLOC. Moreover, the expression of lncRNA TCONS_00040607 correlated negatively with the expression of *AhR*.

### Validation of RNA-Seq data by real-time PCR

**Fig. 6:**
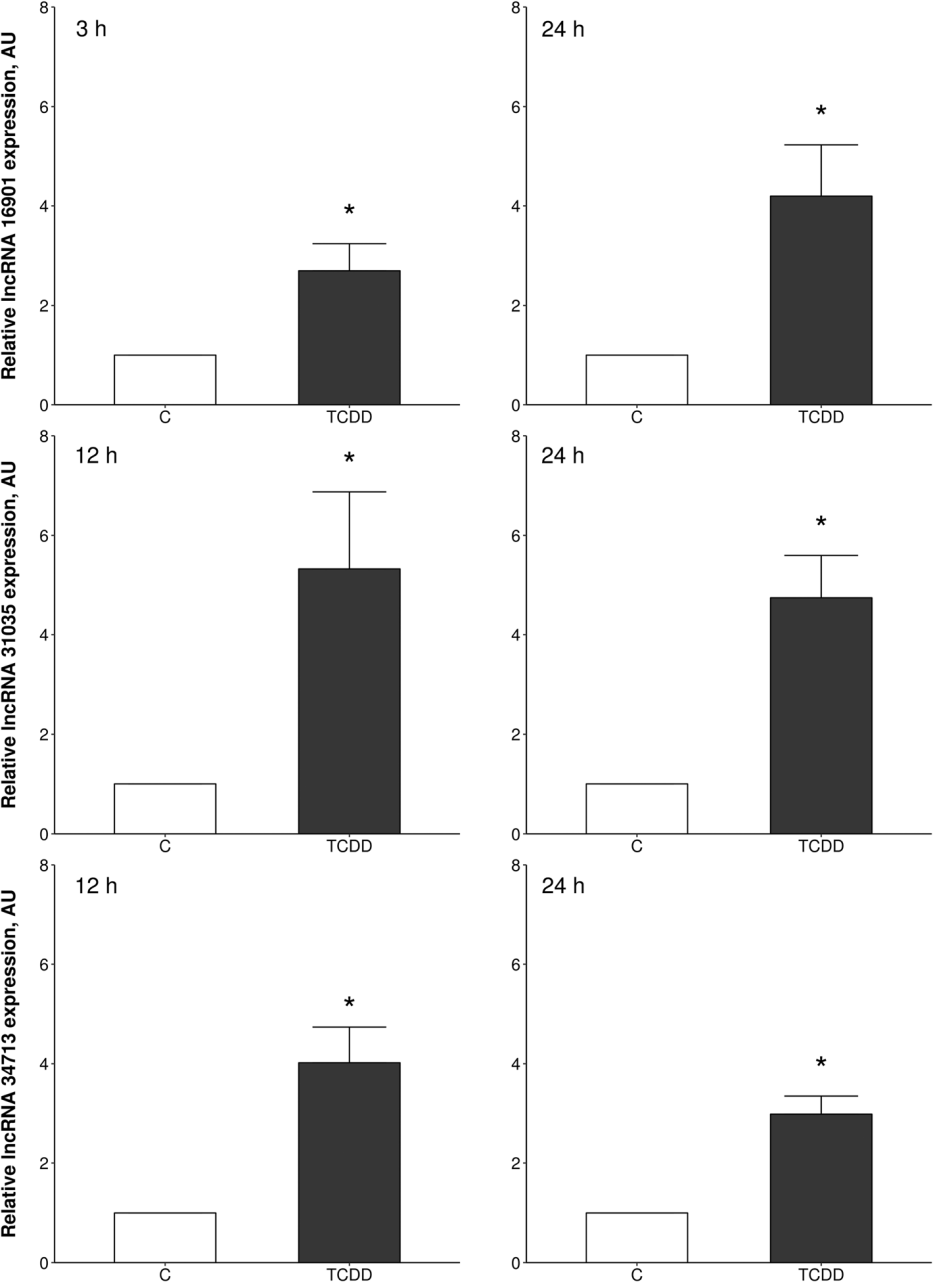
Real-time PCR validation of three selected differentially expressed lncRNAs (TCONS_00016901, TCONS_00031035, TCONS_00034713) which were identified in TCDD-treated porcine granulosa cells (treated vs. untreated cells) by RNA-Seq. Data are expressed as mean ± SEM (*n* = 3–4). Statistical analysis was performed using Student’s *t*-test. Asterisks designate statistical differences (*P* < 0.05). AU: arbitrary units; C: control; TCDD: 2,3,7,8-tetrachlorodibenzo-*p*-dioxin

To validate the RNA-Seq results, three up-regulated lncRNAs, i.e., TCONS_00016901, TCONS_00031035 and TCONS_00034713 were chosen for real-time PCR. The expression of the selected lncRNAs entirely confirmed the results obtained by RNA-Seq (Fig. 6).

## Discussion

Recent advances in sequencing technologies revealed that the genome is widely transcribed, yielding a large number of ncRNAs. In the current study, we investigated the genome-wide lncRNA expression profile of porcine granulosa cells exposed to TCDD. The effects of TCDD on the porcine granulosa cell transcriptome have been previously examined, however the studies have been focused solely on the expression of genes (RNA-Seq [23]; real-time PCR [29]). In comparison to humans and rodents, information concerning the identification and expression of lncRNAs in pigs is limited [24, 50]. To the best of our knowledge, lncRNAs have not yet been identified in porcine granulosa cells and TCDD effects on the lncRNA expression profile have also not been examined.

A total of 1,666 lncRNAs and 42,913 mRNAs were identified in porcine granulosa cells in the present study. The majority of the identified lncRNAs were classified as intergenic lncRNAs. The identified lncRNAs were found to be shorter in comparison to those of mRNAs. In addition, the exon number of lncRNAs was lower and the exon length was longer than those of mRNAs. The features of the identified lncRNAs were similar to those found in other studies [24, 43, 51, 52].

The analysis of the expression profile of lncRNAs in TCDD-treated porcine granulosa cells revealed the presence of 22 DELs. Similarly to previously reported TCDD-induced changes in granulosal gene expression [23], the most of DELs (15 out of 22) were detected after 3 h of TCDD treatment. Since TCDD was shown to induce oxidative stress in the ovary [53, 54], the altered expression of lncRNAs after 3 h of TCDD treatment may be associated with the regulation of the early cellular stress response to TCDD.

Potential functions of DELs identified in porcine granulosa cells were indirectly predicted via searching their target *cis*- and *trans*-regulated protein-coding genes. Screening of genes which were located within 10 kb from DELs enabled us to select 10 *cis-*target genes, but none of them was enriched into GO classification. To identify the *trans*-target genes presumptively regulated by lncRNAs, we correlated the expression levels of DELs and protein-coding genes. A total of 916 negative and 1,143 positive correlations were detected. The GO results of negatively co-expressed genes suggested a possible involvement of DELs in a variety of biological processes such as “intracellular signal transduction”, “response to stimulus” and “negative regulation of biological process”. Interestingly, *AhR* was found among these negatively *trans*-regulated genes. Although the downregulation of *AhR* is a typical response to TCDD, recent reports concerning ncRNAs may provide more details on TCDD mechanism of action. Li et al. [55] demonstrated that miR-203 (microRNA) suppressed the expression of AhR in TCDD-treated human lung and hepatic cells. In the current study, we identified TCONS_00040607 i.e., lncRNA, the expression of which correlated negatively with that of *AhR* after 3 h of TCDD treatment. It is possible that TCONS_00040607 affects the expression of *AhR* and is involved in its negative regulation during the cellular response to TCDD. These results suggest that the regulation of *AhR* by lncRNAs may constitute part of cellular defense mechanism against dioxins. This hypothesis, however, needs additional experimental verification.

Some of the positively co-expressed genes were enriched in GO terms associated with “cellular response to chemical stimulus” and “regulation of signal transduction”. We found that the expression of *CYP1A1*, coding a protein playing an important role in the metabolism of xenobiotics [56], correlated positively with the expression of two DELs (TCONS_00034713 and TCONS_00031305). Due to the fact that TCDD treatment usually induces *CYP1A1* expression, this enzyme is considered to be a molecular marker of TCDD action [57, 58]. In our previous study, the expression of *CYP1A1* increased significantly in a time-dependent manner after 3, 12 and 24 h of porcine granulosa cell incubation with TCDD [23]. We also demonstrated that the TCDD binding to the porcine CYP1A1 active site resulted in a rapid closure of the enzyme substrate channels. This phenomenon may partially explain TCDD’s high resistance to biodegradation [59]. If the TCDD binding causes a continuous CYP1A1 blockage, the cellular response to TCDD may induce an extended synthesis of CYP1A1. The fact that the expression of two DELs: TCONS_00034713 and TCONS_00031305 correlated positively with the expression of *CYP1A1* indicates their supportive role in the cellular reaction to TCDD.

Five DELs (TCONS_00005658; TCONS_00016901; TCONS_00048979; TCONS_00060223; TCONS_00064401) were found to be expressed only in TCDD-treated cells. The expression of two DELs (TCONS_00048979 and TCONS_00060223) correlated negatively with the expression of the same three genes: *ERN1*, *LIMK2* and *EPHB4*. *ERN1* is associated with endoplasmic reticulum stress and ER protein folding [60, 61]. *EPHB4*, in turn, is linked with the proliferation of ovarian carcinoma cells [62] and *LIMK2* with actin microfilament disruption in porcine oocytes [63]. The obtained data imply that TCONS_00048979 and TCONS_00060223 are mediators of TCDD action in porcine granulosa cells.

In addition, we identified three DELs located in the antisense strand of protein coding genes. These antisense strands were found to positively or negatively regulate the expression of their sense counterparts and, therefore, they attracted a lot of attention [64, 65]. In the current study, TCONS_00038918, TCONS_00030731 and TCONS_00064964 were found to be located in the respective antisense strands of *MafF*, *TGFβI* and *NHS*. MafF belongs to the small MAF family of basic-leucin zipper transcription factors, which affect genes encoding proteins responsible for xenobiotic metabolism and antioxidation [66–68]. *TGFβ1* encodes an extracellular matrix (ECM) protein reported to interact with various matrix molecules (collagens, fibronectin and laminin), contributing to cell adhesion, proliferation and migration [69]. ECM proteins were found to be affected by TCDD in marmosets [70]. *NHS* products, in turn, were described to maintain cell morphology by remodeling the actin cytoskeleton [71]. The in silico data concerning the possible relationships between lncRNAs and protein-coding genes in porcine granulosa cells treated with TCDD are supported by the results of our recent study [72]. In this study, the abundance of heat shock proteins as well as cytoskeleton and ECM proteins were significantly affected by TCDD in porcine granulosa cells.

Similarly to protein-coding genes, lncRNAs also undergo alternative splicing, resulting in the formation of numerous lncRNA isoforms and, in consequence, extending their regulatory capabilities [73, 74]. It was demonstrated that xenobiotics may affect alternative splicing, modifying the process in a specific manner [75]. In the current study, the events of differential usage of exons and splice junctions in lncRNAs loci were identified after 3 h and 12 h of cell incubation with TCDD. The majority of the identified events were defined as differentially expressed exons and only a few events were described as splice junctions. It is of interest that two alternatively spliced forms for XLOC_020835 were detected after TCDD treatment. In the current study, the expression of one of these forms i.e., TCONS_00040607, was found to negatively correlate with the expression of *AhR*. These facts implicate that TCONS_00040607 is involved in the regulation of the TCDD-affected *AhR* expression in porcine granulosa cells.

## Conclusions

In the current study, we identified and characterized lncRNAs of porcine granulosa cells. We also examined the effects of TCDD on the lncRNA expression profile. The co-expression analysis revealed that the identified lncRNAs may influence the expression of numerous genes, including those involved in: 1) cellular response to TCDD (e.g., *AhR*), 2) dioxin metabolism (e.g., *CYP1A1, MafF*), 3) endoplasmic reticulum stress (e.g., *ERN1*) as well as 4) adhesion and proliferation of cells (*TGFβ1*, *NHS*, *LIMK2*). Our results suggest that lncRNAs constitute a part of the regulatory apparatus of TCDD action in porcine granulosa cells. This study may provide the foundation for future research focused on molecular effects exerted by TCDD in ovarian cells.

## Additional files

Additional file 1: Summary of the mapping of the RNA sequences to the reference genome. (XLSX 13 kb)

Additional file 2: *Cis*-target genes predicted to be regulated by differentially expressed lncRNAs in porcine granulosa cells exposed to TCDD for 3, 12 and 24 h. (XLSX 50 kb)

Additional file 3: Negatively co-expressed *trans*-target genes predicted to be regulated by differentially expressed lncRNAs identified in porcine granulosa cells exposed to TCDD for 3, 12 and 24 h. (XLSX 78 kb)

Additional file 4: Positively co-expressed *trans*-target genes predicted to be regulated by differentially expressed lncRNAs identified in porcine granulosa cells exposed to TCDD for 3, 12 and 24 h. (XLSX 126 kb)

Additional file 5: Functional enrichment analysis of the negatively co-expresed *trans*-target genes predicted to be regulated by differentially expressed lncRNAs identified in porcine granulosa cells exposed to TCDD for 3, 12 and 24 h. (XLSX 54 kb)

Additional file 6: Functional enrichment analysis of the positively co-expresed *trans*-target genes predicted to be regulated by differentially expressed lncRNAs identified in porcine granulosa cells exposed to TCDD for 3, 12 and 24 h. (XLSX 36 kb)

Additional file 7: Differential expression of exons and the occurence of splice junction in XLOCs of the lncRNAs identified in porcine granulosa cells exposed to TCDD for 3 and 12 h. (XLSX 16 kb)

## Abbreviations

AhR: Aryl hydrocarbon receptor
antisense lncRNAs: LncRNAs originate from the opposite strand of DNA
ARNT: AhR nuclear translocator
bFGF: Fibroblast growth factor-basic human
bHLH-PAS: The basic helix-loop-helix/PER-ARNT-SIM
CNCI: Coding-Non-Coding Index
CPC: Coding Potential Calculator
CYP1A1: Cytochrome P450 1A1
DELs: Differentially expressed lncRNAs
DMEM: Dulbecco’s modified Eagle’s medium
E_2_: Estradiol
EPHB4: Ephrin type-B receptor 4
ERN1: Endoplasmic reticulum to nucleus signaling 1
FBS: Fetal bovine serum
FEELnc: FlExible Extraction of LncRNAs
GO: Gene Ontology database
LIMK 2: LIM domain kinase 2
lincRNAs: LncRNAs originate from intergenic regions of the genome
linRNAs: LncRNAs originate from introns of protein-coding genes
lncRNAs: Long non-coding RNAs
MafF: MAF bZIP transcription factor F
ncRNAs: Non-coding RNAs
NEAA: MEM non-essential amino acid solution
NHS: NHS actin remodeling regulator
nt: Nucleotides
P_4_: Progesterone
PAH: Polycyclic aromatic hydrocarbon
RIN: RNA integrity number
RNA-Seq: RNA sequencing
TCDD: 2,3,7,8-tetrachlorodibenzo-*p*-dioxin
TGFβI: Transforming growth factor beta-induced
